# The Mutational, Epigenetic, and Transcriptional Effects Between Mixed High-Energy Particle Field (CR) and ^7^Li-Ion Beams (LR) Radiation in Wheat M_1_ Seedlings

**DOI:** 10.3389/fpls.2022.878420

**Published:** 2022-05-11

**Authors:** Bo Li, Linshu Zhao, Shuo Zhang, Haiya Cai, Le Xu, Bingzhuang An, Rong Wang, Gang Liu, Yonggang He, Chunhai Jiao, Luxiang Liu, Yanhao Xu

**Affiliations:** ^1^Hubei Key Laboratory of Food Crop Germplasm and Genetic Improvement, Food Crops Institute, Hubei Academy of Agricultural Sciences, Wuhan, China; ^2^Institute of Crop Sciences, Chinese Academy of Agricultural Sciences, Beijing, China; ^3^Hubei Collaborative Innovation Centre for the Industrialization of Major Grain Crops, Yangtze University, Jingzhou, China

**Keywords:** ionizing radiation, genetic variation, mutation distribution, transcriptome sequencing, DNA methylation

## Abstract

Ionizing radiation (IR) is an effective approach for mutation breeding. Understanding the mutagenesis and transcriptional profiles induced by different mutagens is of great significance for improving mutation breeding efficiency. Here, using RNA sequencing and methylation-sensitive amplification polymorphism (MSAP) approaches, we compared the genetic variations, epigenetics, and transcriptional responses induced by the mixed high-energy particle field (CR) and ^7^Li-ion beam (LR) radiation in M_1_ seedlings of two wheat genotypes (Yangmai 18 and Yangmai 20). The results showed that, in both wheat genotypes, CR displayed significantly a higher mutation efficiency (1.79 × 10^–6^/bp) than that by LR (1.56 × 10^–6^/bp). The induced mutations were not evenly distributed across chromosomes and varied across wheat genotypes. In Y18 M_1_, the highest number of mutations were detected on Chr. 6B and Chr. 6D, whilst in Y20 M_1_, Chr. 7A and Chr. 3A had the highest mutations. The transcript results showed that total of 4,755 CR-regulated and 1,054 LR-regulated differentially expressed genes (DEGs) were identified in the both genotypes. Gene function enrichment analysis of DEGs showed that these DEGs overlapped or diverged in the cascades of molecular networks involved in “phenylpropanoid biosynthesis” and “starch and sucrose metabolism” pathways. Moreover, IR type specific responses were observed between CR an LR irradiation, including specific TFs and response pathways. MSAP analysis showed that DNA methylation level increased in LR treatment, while decreased at CR. The proportion of hypermethylation was higher than that of hypomethylation at LR, whereas a reverse pattern was observed at CR, indicating that DNA methylation plays critical roles in response to IR irradiation. All these results support that the response to different IRs in wheat includes both common and unique pathways, which can be served as a useful resource to better understand the mechanisms of responses to different IRs in other plants.

## Introduction

Mutagenesis is a fundamental tool to study gene functions and to create new cultivars in plant breeding. Ionizing radiation (IR), a type of physical mutagen, has been widely used as a powerful mutagen in plant breeding due to its highly effective mutagenic effect (Kazama et al., 2017; Ichida et al., 2019; Jo and Kim, 2019). Heavy-ion beams, as one type of IR, are featured with high linear energy transfer (LET), ranging from 22.5 to 4,000 keV μm^–1^. Therefore, heavy-ion beams can ionize more densely and cause more complex DNA damages, such as double strand breaks and clustered damages, than low-LET IRs, such as gamma rays (0.2 keV μm^–1^) (Kazama et al., 2017; Yang et al., 2019). The repair of these complex DNA damages is often incomplete or even error prone, which causes DNA mutations to be retained and inherited by offspring (Jo and Kim, 2019).

The frequency and spectrum of induced mutations have long been regarded as the crucial factors for the use of mutant populations in breeding (Ichida et al., 2019; Jo and Kim, 2019). With the recent advancement of the next generation sequencing technology, the characteristics of mutagenic effects induced by different types of IRs in plants have attracted abundant researchers’ attention (Kazama et al., 2017; Ichida et al., 2019; Li et al., 2019; Tan et al., 2019; Yang et al., 2019; Hase et al., 2020; Zheng et al., 2020). It has been reported that ^7^Li-ion beams (LR: 83.6 keV μm^–1^) induced a greater number of single base substitutions (SBSs) than short insertions and deletions (InDels) in wheat seedlings (Xiong et al., 2019). A comparative analysis in M_3_ *Arabidopsis* suggested that Ar-ion beams (290.0 keV μm^–1^) induced drastic and complex alterations of chromosomes, while carbon-ion beams with moderate LET values (30.0 keV μm^–1^) often induced SBSs and short insertions and InDels (Kazama et al., 2017). The comparative analyses of the mutations induced by carbon-ion beams (50–107 keV μm^–1^) and gamma rays (0.2 keV μm^–1^) in rice (M_4_–M_6_) have found that and the total mutation numbers induced by gamma rays were more than that of carbon-ion beams (Li et al., 2019; Yang et al., 2019). These studies suggested that the induction of mutations may be associated with LET values.

In addition to the commonly used mutagens as mentioned above, the mixed high-energy particle field (CR) is a complex and new type of IR that simulates secondary cosmic radiation, consisting of a range of high-energy particles, some of which, such as pion, positive and negative electrons, photon and proton, contain a higher level of LET (CR: 1.5 GeV μm^–1^), and may thus be of great interest in crop mutation breeding. However, the mutagenic effect and underlying mechanisms of CR in plants remain unclear.

When IR interacts with organisms, it may directly or indirectly cause DNA lesions. The direct interaction is caused by ions hitting on DNA molecules, while the indirect effect is caused by reactive oxygen species (ROS) aggregation through the radiolysis of water (van de Walle et al., 2016; Gudkov et al., 2019; Choi et al., 2021; Meng et al., 2021). ROS can diffuse through the cells and damage their components, but they are also important signaling molecules in plants responses to stresses, triggering related response mechanisms (Volkova et al., 2019). For example, the acute (8 h) and chronic (10 days) gamma rays treatments significantly increased the hydrogen peroxide (H_2_O_2_) content in rice plants at the tillering stage (Choi et al., 2021). Similar result was also observed in the barley seedlings when exposed to gamma rays (Volkova et al., 2019). The analysis of gamma-ray, cosmic-ray, and carbon-ion beams radiation in rice have shown that genes related to “lipid metabolic process” and “gibberellin metabolic process” are greatly induced by gamma rays, while the stimulus responsive genes were significantly overrepresented in the carbon-ion beams irradiated plants (Hwang et al., 2014). In addition, the cosmic rays induced genes were mainly associated with the molecular function group, including “transcription regulator activity” and “oxidoreductase activity” (Hwang et al., 2014). In duckweed, genes related “mitochondrial electron transport” and “ATP synthesis” were specifically upregulated in response to uranium treatment, while genes involved in “calcium signaling” and “degradation of carbohydrate metabolism” were specifically upregulated responding to gamma rays (Fu et al., 2019). In cowpeas, oxidation-reduction process and proteolysis were significantly enriched in gamma rays treatment, while organic substance metabolic process and cellular metabolic process were enriched in proton-beam treatment (Kang et al., 2021). These studies imply that the gene expression of plants in response to the different LET IRs were highly diverse and complex. The target plants are generally with a small genome, ranging from ∼400 Mb (rice) to ∼520 Mb (cowpeas). To date, there are few studies on the mutagenesis effects of IRs on plants with large and complex genome.

DNA methylation is an important epigenetic mechanism that plays crucial roles in maintaining genome stability and adaptation to environmental stresses (Zhang et al., 2018). However, very controversial results have been found through time (Shi et al., 2014; Georgieva et al., 2017; Volkova et al., 2018; Zhao et al., 2018; Marfil et al., 2019). Pine trees (Volkova et al., 2018) and soybean seedlings (Georgieva et al., 2017) from sites contaminated by the chernobyl accident showed an increase in global DNA methylation, while *Arabidopsis* plants from chernobyl radio-contaminated regions showed a decrease in methylation (Horemans et al., 2018). Furthermore, low-dose heavy-ion radiation induced higher proportion of hypermethylation than hypomethylation in rice at CG sites (0.01, 0.2, or 1 Gy), while the opposite was observed at high-dose heavy-ion radiation (2, 5, or 20 Gy) (Zhao et al., 2018). These studies indicate DNA methylation might play an important role in the adaptive responses to IR irradiation. However, DNA methylation remodeling caused by different types of IRs in plants is not clear.

Wheat (*Triticum aestivum* L.) is the most widely cultivated food crop throughout the world, with a large and complex hexaploidy genome (∼16 Gb) (Appels et al., 2018). In the past decades, several mutagens have been used in wheat radiation research and breeding, such as ethyl methanesulfonate (Chen Z. et al., 2020), gamma rays (Bhat et al., 2020), laser (AlSalhi et al., 2018), electron beam (Wang et al., 2019), spaceflight (Xiong et al., 2017), and ^7^Li-ion beam (Xiong et al., 2019). However, most of these studies focused on effects of radiation on physiology, biochemistry, morphology, and stress resistances. There are few researches on mutagenesis effect and radiation responses in wheat M_1_ generation.

For these reasons, we compared mutagenic effects and transcriptional responses induced by two types of IRs with different LETs: ^7^Li-ion beam (LR: 83.6 keV μm^–1^) and the mixed high-energy particle field (CR: 1.5 GeV μm^–1^), in two wheat genotypes (Yangmai 18 and Yangmai 20) at M_1_ seedlings by using RNA-seq. In addition, the epigenetic regulation of DNA methylation in response to different IR treatments were studied using methylation-sensitive amplification polymorphism (MSAP). This study provides the genomic and epigenetic clues for understanding of mutagenesis mechanism induced by LR and CR and the molecular basis for high LETs mutation breeding in wheat.

## Materials and Methods

### Plant Materials and Radiation Treatments

Two wheat genotypes of Yangmai 18 (Y18) and Yangmai 20 (Y20) were used for this study. The dry seeds of Y18 and Y20 were exposed to ^7^Li-ion beam (Y18LR and Y20LR: 83.6 keV μm^–1^) and the mixed high-energy particle field (Y18CR and Y20CR: 1.5 GeV μm^–1^) with the same dose of 100 GY. The irradiation treatments were performed by China Institute of Atomic Energy. The irradiated and untreated (wide type, WT) seeds were placed in plastic germination boxes (13 cm × 19 cm × 9 cm) containing four layers of moistened filter paper with sterile water (100 seeds/each box) and grown in a growth chamber with a 14/10 h and 24/20°C day/night light and temperature cycle. The germination rates were recorded after 3 days. After 7 days of growth, the seedling lengths were measured for 20 plants per replicate. Besides, the plant leaves of each treatment were randomly collected and mixed with more than 20 individuals as one biological replicate, frozen in liquid nitrogen immediately and stored at −80°C. Three biological duplicates of each treatment were set.

### RNA Isolation, Library Construction, and Sequencing

Total RNA of each sample was isolated using the RNA plant Plus Reagent Kit (TIANGEN, China). The quantity and concentration of each RNA sample were determined by NanoDrop 2000 (Thermo Fisher Scientific, United States). The integrity and purification of RNA samples were qualified using the 2100 Bioanalyzer instrument (Agilent Technologies, United States). Library preparation for RNA-seq was conducted using a MGIEasy mRNA Kit (MGI, China) according to manufacturer’s protocol. Finally, 18 cDNA libraries were sequenced on MGISEQ 2000 platform (GOOALGENE, China) and 150 bp pair-end reads were generated.

### Reads Mapping and Assembly

Raw reads produced by the sequencer were filtered to remove reads with low quality using Fastp (version 0.19.7) (Chen et al., 2018) and were further assessed for quality of using FASTQC toolkit (v0.11.9) (Brown et al., 2017). The clean reads of each sample were mapped to the wheat reference genome^1^ using HISAT2 (v2.1.0) (Kim et al., 2015).

### Genetic Mutations Identification

The uniquely aligned reads were used to identify SBSs and InDels variations between the assembled reads and the reference genome sequence using GATK2 software.^2^ To obtain reliable mutations of each mutagenesis progeny, the heterozygous sites of wild type lines were removed in the following analysis. The total numbers of heterozygous and homozygous InDels, SBSs, and different types of SBSs, including transitions (Ti: purine > purine or pyrimidine > pyrimidine) and transversions (Tv: purine > pyrimidine or pyrimidine > purine), were counted for each replicate. Mutation rate was calculated as the average number of mutations per mutant divided by the average length (number of bases) of all genomic regions (Li et al., 2019). The frequency of mutations in every 10-Mb region of each chromosome was calculated and visualized by using Circos software.

### Differentially Expressed Gene Analysis and Functional Annotations

The expression level of each gene was estimated by fragments per kilobase of transcript per million fragments mapped (FPKM). The differentially expressed genes (DEGs) between control and irradiated samples were identified using DESeq2 R package (Anders and Huber, 2010). The cutoff of DEGs were defined by using the standard as | log_2_^(fold change)^ | ≥ 2 and *P*-value ≤ 0.05. Gene Ontology (GO) enrichment analysis of the DEGs was performed using singular enrichment analysis tool with FDR < 0.05 by agriGO v2.0, which assigned all DEGs into three principal categories, namely cellular component, molecular function, and biological process (Tian et al., 2017). Kyoto Encyclopedia of Genes and Genomes (KEGG) enrichment analysis were performed on the OmicShare platform^3^ and significant enrichment were selected at *P* < 0.05. Transcription factors (TFs) were predicted and classified into different families using the PlantTFDB.^4^ Venn Diagrams comparing the number of DEGs across different IR treatments were created using jvenn.^5^ Heatmaps of the gene expressions were illustrated by using TBtools (Chen C. et al., 2020).

### Quantitative Real-Time PCR Analysis

The total RNA of all 18 samples was used for the transcriptome analysis and was also used to make cDNas for quantitative real-time PCR (qRT-PCR) validation. The synthesis of first-strand cDNA was conducted using 1 μg of total RNA from each sample with an UEIris RT mix with Dnase (All-in-One) kit (US Everbright, China) according to the manufacturer’s instructions. qRT-PCR was performed using 2× SYBR Green qPCR Master mix (S2014, US Everbright, China) on a QuantStudio™ 7 Flex Real-Time PCR System (Applied Biosystems, United States). Gene-specific primers for qRT-PCR were designed *via* primer premier 5 and are listed in Supplementary Table 1. qRT-PCR was conducted in triplicate (technical repeats) with three biological replicates for each sample, and the relative gene expression levels were calculated using the 2^–ΔΔCt^ method.

### Methylation-Sensitive Amplification Polymorphism Analysis

The total genomic DNA of samples mentioned above were isolated using modified CTAB method (Doyle and Doyle, 1987). The MSAP procedure is performed to investigate global DNA methylation changes according to an established protocol (Tang et al., 2022). Two restriction enzyme combinations, *Eco*RI/*Hpa*II and *Eco*RI/*Msp*I (Thermo Fisher Scientific, United States), were used for digestion. After ligated to the *Hpa*II/*Msp*I adapter and *Eco*RI adapter, two consecutive PCRs were carried out to produce a specific DNA fragment fingerprint. Twenty primer combinations with three selective nucleotides for the *Eco*RI ends and two to four selective nucleotides for the *Hpa*II/*Msp*I ends were used (Supplementary Table 2). The PCR products were separated by fragment analyze automated CE system (AATI, United States) with the Quick Start Guide 96 Capillary DNF-900 dsDNA Reagent Kit, 35–500 bp (AATI, United States) (Li et al., 2020). The MSAP profiles showing reproducible results between replicates, ranging from 100 to 500 bp, were scored and transformed into a 1/0 binary matrices, where 1 indicates the presence and 0 the absence of a given fragment. Four types of MSAP bands were defined as non-methylation, hemi-methylation, and full-methylation (Table 2) according to Tang et al. (2022).

### Statistical Analysis

The germination and seedling height data were statistically analyzed by one-way ANOVA (SNK methods) with a significance level of *P* < 0.05 using SPSS 18.0 statistical software. The heatmap of gene expression was constructed basing on the Log2FC (RNA-seq) and 2^–ΔΔ*Ct*^ (qRT-PCR) value. The linear correlation coefficient between RNA-seq and qRT-PCR results was detected by using Excel 2010.

## Results

### CR and LR Irradiation Induced Changes in Germination Rate and Seedling Height

**FIGURE 1:**
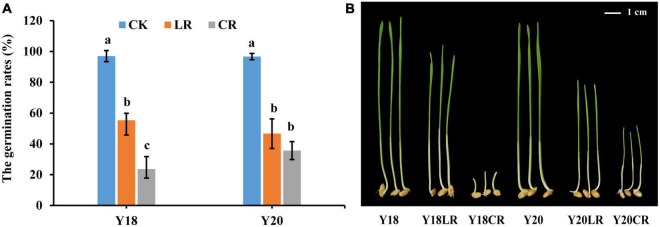
The damage effects of LR and CR on wheat seedlings. **(A)** The germination rates of control and irradiated wheat seeds. **(B)** The height of seedlings germinated from un-irradiated and irradiated wheat seeds grown for 7 days. All treatments were set three biological and three technical replications.

Under control condition, the germination rates for Y18 and Y20 were 97.67 and 96.33%, respectively. Both CR and LR irradiation treatments significantly reduced the seed vitality. The germination rates were significantly reduced to an average of 29.67% (23.67% in Y18CR and 35.67% in Y20CR) under CR (*P* < 0.05), and 51.00% (55.33% of Y18LR and 46.67% of Y20LR) under LR when compared with WT (Figure 1A), suggesting the damaging effect induced by CR is much higher than that induced by LR.

The seedlings of the two wheat genotypes displayed comparable height in the control condition at 7 days after germination (8.57 and 8.1 cm for Y18 and Y20, respectively). The seedling height of both genotypes were significantly reduced under both LR and CR treatments. For both genotypes, seedlings of CR treatment exhibited much more suppressed with an average seedling height reduction of 57.05% compared to the control (1.18 and 3.10 cm for Y18CR and Y20CR, respectively), whilst seedlings with LR treatment only have an average height reduction of 28.45% (6.95 and 5.13 cm for Y18LR and Y20LR, respectively) (Figure 1B). Interestingly, the two wheat genotypes showed clearly different sensitivity to the two types of IR irradiation. In particular, Y18 (18.87% height reduction) displayed to be less sensitive than Y20 (36.63%) to LR treatment. In contrast, Y18 growth was much more severely suppressed (86.19% height reduction) by CR treatment than Y20 (61.73% height reduction) (Figure 1B).

### Identification of Single Base Substitutions and Insertions and Deletions Induced by CR and LR in Wheat M_1_ Seedlings

To identify the mutations caused by IR treatments, a total of 18 cDNA libraries (Supplementary Table 3) were constructed for sequencing. After a stringent quality filtering process, an average of 7.60 Gb high-quality clean data (Q30 > 89.49% and uniquely mapped rates > 83.92%) was retained for each sample, which represents ∼57 folds of the total length of the all predicted high-confidence genes in wheat (Supplementary Table 3). Correlation heat map analysis detected high correlations between the biological replicates (Supplementary Figure 1). These results indicated the overall reproducibility and quality of the assay.

To compare the mutagenic effects of LR and CR on wheat, SBSs and InDels between M_1_ and WT seedlings were detected. An average of 24,321 mutations were detected for each genotype under LR and CR treatments (Table 1). The number of SBSs are much higher than that of InDels in all samples, accounting 87.46–89.59% of the total mutations. In both wheat genotypes, CR treatment induced higher number of mutations (average 26,002 mutations) and mutation rate (1.79 × 10^–6^/bp) than LR (average 22,641 mutations and mutation rate 1.56 × 10^–6^/bp) (Table 1). LR induced an average of 11,432 heterozygous SBSs, which was higher than homozygous SBSs (8,396). Whilst the number of homozygous SBSs (average of 12,108) induced by CR is higher than that of heterozygous SBSs (10,985). Similarly, LR induced a higher number of heterozygous InDels (average of 1,680) than that of homozygous InDels (1,135), whereas the number of homozygous (average of 1,438) and heterozygous (1,472) InDels are comparable under CR treatment (Table 1).

**TABLE 1:** Numbers of mutations induced by LR and CR.

Samples	Total	SNPs		InDels		Mutation rates	Ti/Tv ratio
		Homozygous	Heterozygous	Homozygous	Heterozygous		
Y20LR	22,720	8,454	11,467	1,108	1,691	1.56 × 10^–6^	1.95 (13,166/6,755)
Y20CR	25,033	11,754	10,269	1,483	1,527	1.72 × 10^–6^	1.92 (14,481/7,542)
Y18LR	22,563	8,338	11,396	1,161	1,668	1.55 × 10^–6^	1.94 (13,029/6,705)
Y18CR	26,971	12,462	11,700	1,393	1,416	1.85 × 10^–6^	1.85 (15,697/8,469)

**TABLE 2:** Methylation-sensitive amplification polymorphism-based cytosine methylation levels in wheat seedlings under WT, LR, and CR treatments.

MSAP band types	Patterns^a^		Y20			Y18		
			
	*Hpa*II	*Msp*I	WT	LR	CR	WT	LR	CR
I	1	1	84	73	94	80	64	99
II	1	0	74	74	92	70	82	90
III	0	1	156	144	126	169	135	141
IV	0	0	69	91	71	64	102	53
Hemi-methylated ratio (%)^b^			19.32	19.37	24.02	18.28	21.41	23.50
Full methylated ratio (%)^c^			58.75	61.52	51.44	60.84	61.88	50.65
Total methylated ratio (%)^d^			78.07	80.89	75.46	79.11	83.29	74.15

*^a^The symbol “1” or “0” represents the presence or absence of bands, respectively. Type I (HpaII/MspI, 11) indicates unmethylation, type II (HpaII/MspI, 10) indicates hemi-methylation, type III (HpaII/MspI, 01), and type IV (HpaII/MspI, 00) indicate full methylation.*

*^b^Hemi-methylated ratio (%) = [(II/(I + II + III + IV)] × 100.*

*^c^Fully methylated ratio (%) = [(III + IV)/(I + II + III + IV)] × 100.*

*^d^Total methylated ratio (%) = [(II + III + IV)/(I + II + III + IV)] × 100.*

Among the SBSs in all target samples, six possible types of substitution were detected. Ti mutations (purine to purine or pyrimidine to pyrimidine, including A/T to G/C and G/C to A/T) (average 65.78% of all SBSs) is much higher than Tv mutations (average 34.22% of all SBSs) (purine > pyrimidine or pyrimidine > purine, including A/T to C/G, A/T to T/A, G/C to T/A, and G/C to C/G) in the all samples. The ratio of Ti/Tv ranges from the lowest (∼1.89) in Y18CR to the highest (∼1.95) in Y20LR (Table 1).

### Chromosome Distribution of Mutants Induced by CR and LR in Wheat M_1_ Seedlings

**FIGURE 2:**
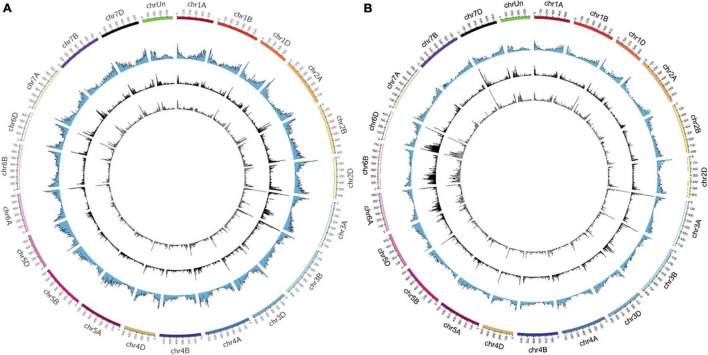
The distribution of SBSs and InDels on the 21 chromosomes identified from IR-irradiated wheat. **(A,B)** Represent the varieties of Y20 and Y18 expose to LR and CR, respectively. The variants in the circle from outside to inside were chromosomes, gene density, LR-induced, and CR-induced mutations, respectively.

The distribution of the identified mutations (SBSs and InDels) on wheat chromosomes were shown in Figure 2. As a result, CR induced mutations were ranged from 627 (chr4D) to 2,728 (chr6B) and 664 (chr4D) to 1,940 (chr3A) in Y18CR and Y20CR, respectively. On the other hand, LR-induced mutations were ranged from 533 (chr4D) to 2,269 (chr6B) and 626 (chr4D) to 1,705 (chr7A) in Y18LR and Y20LR, respectively. In Y18 and Y20 M_1_ seedlings, CR induced an average of 1,226 (Y18CR) and 1,138 mutations (Y20CR) in each chromosome, while LR induced an average of 1,026 (Y18LR) and 1,033 (Y20LR) mutations in each chromosome. Most of the mutations in LR and CR irradiated wheat seedlings were found on the terminal regions of chromosomes in our study (Figure 2). Furthermore, the highest mutation number in Y18 M_1_ (Y18LR and Y18CR) seedlings were detected at chr6B [accounting for 10.06% (2,269) and 10.11% (2,728) of total mutations, respectively] and chr6D [accounting for 9.83% (2,219) and 10.01% (2,701) of total mutations, respectively]. Instead, for mutations in Y20 M_1_ seedlings, Chr. 7A [accounting for 7.50% (1,705) and 6.70% (1,676) in Y20LR and Y20CR, respectively] and chr3A [accounting for 6.30% (1,432) and 7.75% (1,940) in Y20LR and Y20CR, respectively] seem to be more prone to mutations (Figure 2).

We then investigated the frequencies of mutations (SBSs and InDels) in per 10 Mb regions of wheat genome. The result showed that a highest frequency mutation region, locating on 10–15 Mb of chr3A, was commonly detected in Y18CR and Y20LR, accounting for 0.55 and 0.70% of the total mutations, respectively. Instead, the highest frequency mutation regions of Y18LR and Y20CR were located on 470–473.59 Mb of chr6D and 5–10 Mb of chr3B, accounting for 0.44 and 0.62% of the total mutations, respectively (Figure 2).

### Identification of Differentially Expressed Genes Induced by CR and LR in Wheat Seedlings

**FIGURE 3:**
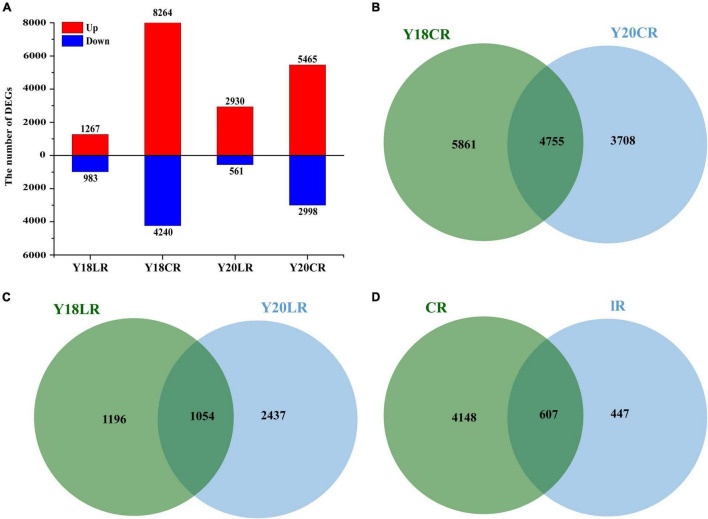
Summary of the differentially expressed genes of Y18 and Y20 under CR and LR treatments. **(A)** A summary of the numbers of up- and down-regulated DEGs. Venn map of differentially expressed genes in two genotypes of wheat under CR **(B)** and LR **(C)** treatment. **(D)** Venn map of overlapped differentially expressed genes between LR and CR treatments in both genotypes.

To analyze the transcriptional changes related to IRs responses, we compared the transcriptomes of Y20 and Y18 M_1_ seedlings exposed to LR and CR to the control without IRs treatment. The results showed that the number of DEGs varied across IR types and wheat genotypes (Figure 3A). In both wheat genotypes, LR resulted in relatively smaller numbers of DEGs (2,250 in Y18LR and 3,491 in Y20LR) than CR (Y18CR 10,618 and Y20CR 8,463). The number of upregulated DEGs were significantly higher than the downregulated DEGs in all samples, with the proportion of up-regulated DEGs ranged from 56.31% (Y18LR) to 83.93% (Y20LR) (Figure 3A).

The cross-comparison between two wheat genotypes Y18 and Y20 showed that most of the IRs induced DEGs were genotype specific. About 41.99% (4,755) DEGs induced by CR were overlapped between Y18 and Y20, while, only 22.49% (1,054) DEGs induced by LR were overlapped between Y18 and Y20 (Figures 3B,D). Of these overlapped DEGs, 4,148 (79.74%) and 447 (8.59%) DEGs were exclusively expressed in response to CR and LR, respectively (Figure 3D), whilst the rest 607 DEGs (11.67%) was responsive to both CR and LR treatments in both genotypes, implying specific and common regulatory changes in wheat responses to different types of IR (Figure 3D).

### Functional Analysis of the LR and CR Induced Differentially Expressed Genes

To further dissect the differential response to IR treatments, the 4,775 CR commonly regulated and 1,054 LR commonly regulated DEGs were used for GO enrichment analysis. Of the 4,755 CR co-expressed DEGs, 65 GO terms were significantly enriched, of the 1,054 LR co-expressed DEGs, 19 GO terms were significantly enriched. Interestingly, most of the enriched GO terms in LR (15) were overlapped with those under CR. These overlapped GOs include nine biological processes (“response to stimulus,” “carbohydrate metabolic process,” “response to abiotic stimulus,” “response to stress,” “response to endogenous stimulus,” “secondary metabolic process,” “response to biotic stimulus,” “response to external stimulus,” and “biosynthetic process”) and nine cellular components (“extracellular region,” “external encapsulating structure,” “cell wall,” “plasma membrane,” “membrane,” and “vacuole”) (Supplementary Table 4).

**FIGURE 4:**
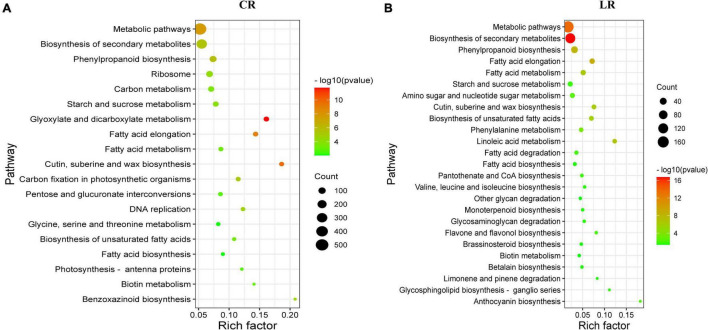
Scatterplot of enriched KEGG pathways for differentially expressed genes under CR **(A)** and LR **(B)** irradiation. The rich factor is the ratio of the DEG number to the total gene number in a certain pathway. The size and color of the dots represent the gene number and the range of the –log10 (*P*-value), respectively.

The KEGG pathway enrichment analysis showed that, a total of 19 and 25 pathways were significantly enriched under CR and LR irradiation, respectively (Figure 4). Among these pathways, 10 common pathways were observed in both IR responses, which include “biosynthesis of secondary metabolites,” “metabolic pathways,” “fatty acid elongation,” “phenylpropanoid biosynthesis,” “cutin, suberine, and wax biosynthesis,” “biosynthesis of unsaturated fatty acids,” and “starch and sucrose metabolism.”

In addition to the above common pathways, nine pathways including “glyoxylate and dicarboxylate metabolism,” “carbon fixation in photosynthetic organisms,” “DNA replication,” “carbon metabolism,” “pentose and glucuronate interconversions,” and “photosynthesis – antenna proteins,” were uniquely enriched under CR treatment (Figure 4A), and 15 pathways were uniquely enriched under LR treatment, including “phenylalanine metabolism,” “amino sugar and nucleotide sugar metabolism,” “anthocyanin biosynthesis,” and “flavone and flavonol biosynthesis” (Figure 4B). Taken together, these results highlighted the involvement of both common and unique pathways in LR and CR treatments responses.

### Phenylpropanoid Biosynthesis and Antioxidant Involvement in Response to CR and LR

**FIGURE 5:**
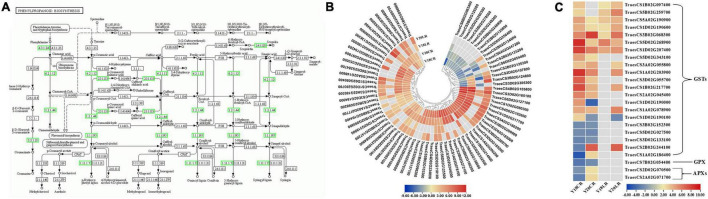
Differentially expressed genes involved in the phenylpropanoid biosynthesis pathway and ROS scavenging process. **(A)** The location of DEGs in phenylpropanoid biosynthesis pathway. The expression pattern of the 73 peroxidase **(B)** and ROS related genes **(C)**.

Among the detected DEGs, a total of 101 genes related to phenylpropanoid biosynthesis were found to be differentially expressed under CR and LR treatments (Figure 5A and Supplementary Table 5). The 101 phenylpropanoid related genes included 73 peroxidases, 10 beta-glucosidases, 9 phenylalanine ammonia-lyases, 3 cinnamoyl-CoA reductases, 2 4-coumarate-CoA ligases, 2 scopoletin glucosyltransferases, 1 shikimate *O*-hydroxycinnamoyl transferase, and 1 cinnamyl alcohol dehydrogenase (Figure 5A and Supplementary Table 5). Of the phenylpropanoid related genes, 21 genes were co-regulated by LR and CR, including 5 phenylalanine ammonia-lyases and 16 peroxidases (Supplementary Table 5). Among the phenylpropanoid related genes, 3 phenylalanine ammonia-lyase and 2 peroxidase genes were genotype specific and mainly upregulated in Y20 under CR and LR treatments, while downregulated in Y18 under CR and LR treatments. Moreover, 34 phenylpropanoid related genes, including 1 phenylalanine ammonia-lyase, 1 cinnamoyl-CoA reductase, 1 cinnamyl alcohol dehydrogenase, 5 beta-glucosidases, 2 scopoletin glucosyltransferases, and 24 peroxidases, were exclusively expressed under CR irradiation. Noteworthy, most of the 73 peroxidase genes were found significantly upregulated at one or both IR treatments (Figure 5B and Supplementary Table 5). In addition, 4 DEGs encoding 1 4-coumarate–CoA ligase, 1 beta-glucosidase, and 2 peroxidases were only differentially expressed under LR irradiation (Supplementary Table 5).

Moreover, 24 genes encoding enzymes involved in ROS metabolism were also differentially expressed, mainly including glutathione *S*-transferase (GST), ascorbate peroxidases (APX), and glutathione peroxidase (GPX) (Figure 5C). Twenty-one GSTs were differentially expressed, and over half of them were up-regulated under LR or CR treatments. In which, 4 GSTs were co-upregulated at both IR treatments in two genotypes, while 3 and 3 GSTs were specifically up- and down-regulated by CR treatment, respectively. Additionally, 2 APXs and 1 GPX were only induced by CR treatment.

### Identification of Differentially Expressed Genes Related to Starch and Sucrose Metabolism in Response to CR and LR

**FIGURE 6:**
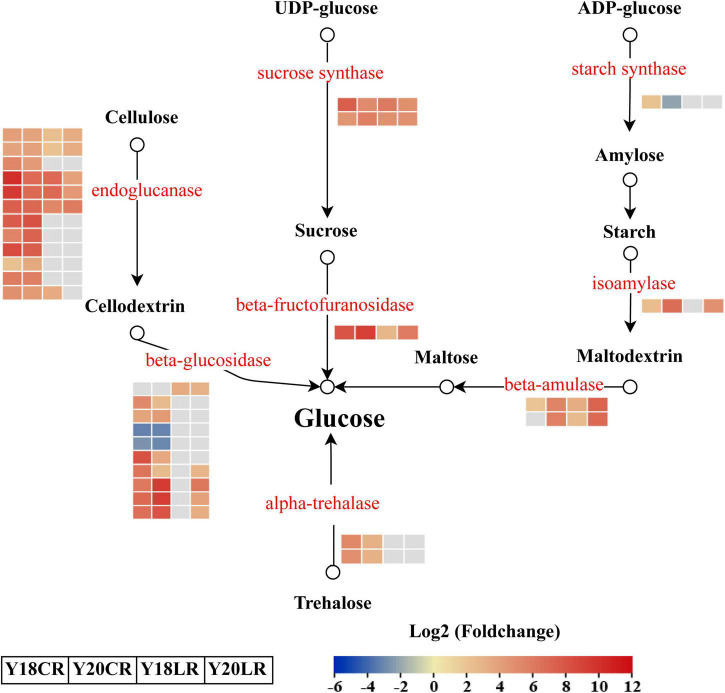
Differentially expressed genes involved in the starch and sucrose metabolism pathways in response to CR and LR irradiation. Red letters represent DEGs and the heatmap represent the expression of the corresponding DEGs induced by CR and LR irradiation. The rectangle filled in red, blue, and gray represent the upregulated, downregulated, and non-different genes in the pathway after irradiation, respectively. Black arrow indicated a direct product.

In addition to the phenylpropanoid biosynthesis pathway, KEGG enrichment results also showed that genes related to starch and sucrose metabolism were enriched significantly in both LR and CR treatments (Figure 4). A total of 31 DEGs were identified involved in four significant metabolic processes in “starch and sucrose metabolism,” which include “cellulose degradation” (22), “trehalose degradation” (2), “sucrose degradation” (3), and “starch degradation” (4) (Figure 6 and Supplementary Table 6). In the “sucrose degradation” process, 3 DEGs (2 sucrose synthases and 1 beta-fructofuranosidase) were significantly upregulated in the both IR treatments, while the DEGs for “cellulose degradation” and “trehalose degradation” processes were mainly upregulated under CR treatment. Notably, one beta-amulase gene involved in “starch degradation” process was co-upregulated in both IR treatments, while the expression abundance was higher in Y20 than that in Y18 under both CR and LR treatments (Figure 6 and Supplementary Table 6).

### Identification of Transcription Factors in Response to CR and LR

**FIGURE 7:**
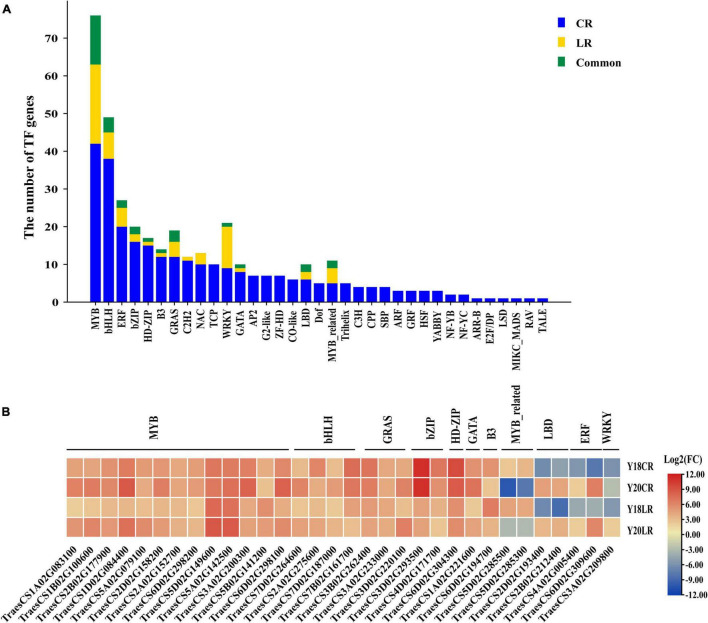
Number and classification of TF genes among the CR and LR induced DEGs **(A)**. Heat map constructed based on log2 (fold change) of common TFs **(B)**.

Transcription factors play crucial functions in stress adaptive signaling cascades and control the expression of numerous genes. A total of 285 TF-encoding DEGs (35 TF families) were found for CR irradiation, whilst only 63 TF-encoding DEGs (13 TF families) were found for LR irradiation (Figure 7A). The most abundant TF family is MYB (42 and 21 for CR and LR, respectively) family, followed by bHLH (38 and 7), ERF (20 and 5), and bZIP (16 and 2) (Figure 7A), sequentially. A total of 32 TF-encoding DEGs (11 TF families) were commonly detected under both IR treatments, including MYB (13), bHLH (4), GRAS (3), bZIP (2), MYB_related (2), HD-ZIP (1), GATA (1), B3 (1), LBD (2), ERF (2), and WRKY (1) (Figure 7B). Among these common TFs, majority were co-upregulated in both genotypes under LR and CR treatments (Figure 7B), with the exception of 2 MYB_related, 2 LBD, and 2 ERF TFs, which were down-regulated in CR and LR (Figure 7B). In addition, 190 TFs belonging to 35 families were induced only by CR treatment, such as TCP (10), AP2 (7), G2-like (7), Trihelix (5), HSF (3), and ARF (3). While 4 TFs, including 1 bHLH, 1 WRKY, and 2 MYB genes were specifically induced by LR (Supplementary Table 7).

### Quantitative Real-Time PCR Validation

To verify the accuracy of our RNA-seq, 15 DEGs in phenylpropanoid biosynthesis, starch and sucrose metabolism, and TFs were randomly selected (5 of each) for qRT-PCR validation. The results showed that the expression patterns were generally consistent with the RNA-seq data, suggesting that the high accuracy and reproducibility of our RNA-seq data (*R*^2^ = 0.9011) (Supplementary Figure 2).

### Global DNA Methylation Changes Induced by CR and LR Treatments

Cytosine methylation patterns in the leaves of two wheat genotypes under IR treatments were detected using 20 pairs of primers. An average of 383 clear and reproducible bands were detected (Table 2 and Supplementary Figure 3). Most of the CCGG sites were shown to be largely methylated with the values ranging between 74.15 and 83.29%. In the control, we observed slight variations in the numbers of methylated sites between the Y20 (78.07%) and Y18 (79.11%) genotypes. Compared with control, LR displayed increased DNA methylation levels in both genotypes (83.29 and 80.89% in Y18LR and Y20LR, respectively), while CR-treated samples decreased in the methylation levels (75.46 and 74.15% in Y20CR and Y18CR, respectively). Further analyses showed that fully methylated bands were more predominant than the hemi-methylated ones.

To further investigate the difference of wheat DNA methylation in response to IR treatments, 16 possible banding patterns between control and IR treatments were identified and classified into three groups: no change, hypomethylation, and hypermethylation. Results (Table 3) showed that ∼47.96% of the CCGG sites remained unchanged under IR treatments. The average percentage of hypomethylated bands was 28.85% under CR treatment, higher than that of LR treatment (23.45%). In contrast, the average percentage of hypermethylated bands was 21.36% under CR treatment, lower than that of LR treatment (30.43%) (Table 3). Additionally, different levels of DNA methylation patterns between Y18 and Y20 were also observed under both IR treatments. For example, the hypomethylation level in Y18CR was higher than that for Y20CR, while more hypomethylation events under LR occurred in Y20LR than Y18LR.

**TABLE 3:** Alternations of DNA methylation patterns induced by CR and LR treatments.

Description of patterns	WT		IR-treated		Y18CR	Y20CR	Y18LR	Y20LR
	*Hpa*II	*Msp*I	*Hpa*II	*Msp*I				
No change	1	1	1	1	46	46	40	36
	0	0	0	0	3	21	13	17
	1	0	1	0	45	36	31	24
	0	1	0	1	91	85	90	93
				Total	185 (49.73%)	188 (49.87%)	174 (46.9%)	170 (45.33%)
Hypomethylation	1	0	1	1	9	11	6	11
	0	1	1	1	28	33	7	12
	0	0	1	1	16	4	12	14
	0	1	1	0	13	13	16	19
	0	0	1	0	17	21	25	13
	0	0	0	1	28	23	15	25
				Total	111 (29.84%)	105 (27.85%)	81 (21.83%)	94 (25.07%)
Hypermethylation	0	1	0	0	37	25	55	32
	1	0	0	0	5	21	21	31
	1	1	0	0	8	4	13	11
	1	1	0	1	11	12	17	18
	1	1	1	0	15	22	10	19
				Total	76 (20.43%)	84 (22.28%)	116 (31.27%)	111 (29.6%)

*The symbol “1” or “0” represents the presence or absence of bands, respectively. Type I (HpaII/MspI, 11) indicates unmethylation, type II (HpaII/MspI, 10) indicates hemi-methylation, type III (HpaII/MspI, 01), and type IV (HpaII/MspI, 00) indicate full methylation.*

## Discussion

Morphometric parameters are often considered as integral indicators of the plant response to IR (Gudkov et al., 2019). The carbon-ions irradiated eye bean seeds showed a significant decrease of germination rate and seedling height, while no difference observed between Ti-ions irradiated seeds and non-irradiated seeds (De Micco et al., 2021). Wang et al. (2018) observed that the growth parameters of germination, root length, and fresh weight in *Arabidopsis* were decreased by high-dose carbon-ion beams (100–200 Gy). A previous study on gamma rays irradiation in barley M1 plants found that the lengths and weights of 100 Gy-irradiated roots and shoots were significantly lower than the control (Volkova et al., 2019). Consistently, we showed that both IR radiations significantly reduce the germination rates and seedling growth, suggesting the inhibitory effect on plant growth and development are dependent on the type of IR (De Micco et al., 2021).

### Higher Frequency of Mutations Induced by CR and LR

Ionizing radiation has been considered the most powerful source of mutagenesis for improving agricultural traits in various crops worldwide. The mutation spectrum and frequency are important factors during the selection of appropriate mutagens for mutation breeding and gene functional mutation (Kazama et al., 2017; Ichida et al., 2019; Jo and Kim, 2019; Li et al., 2019). The mutation frequencies in the genome of M_2_ rice plants generated by gamma rays and carbon-ion beams irradiations were estimated to be 3.2 × 10^–8^ and 2.4 × 10^–8^/bp, respectively (Li et al., 2019). While the carbon-ion beams induced mutation frequencies reported by Yang et al. (2019) and Oono et al. (2020) were average 2.4 × 10^–7^/bp (M_2_) and 2.7 × 10^–7^/bp (M_2_ and M_3_), respectively. More recently, proton beams and gamma rays induced mutation frequencies of M_2_ mutants in rice were estimated ∼5.0 × 10^–7^ and ∼7.0 × 10^–7^/bp, respectively (Lee et al., 2021). In *Arabidopsis*, mutation rates induced by gamma rays (M_2_–M_6_) and carbon-ion irradiation (M_2_) were 1.4–2.2 × 10^–7^ and 0.9–1.2 × 10^–7^/bp (Hase et al., 2020). However, the mutation rates obtained in the present study were higher than those estimated by these previous studies (Li et al., 2019; Yang et al., 2019; Hase et al., 2020; Oono et al., 2020; Lee et al., 2021). The present study demonstrates that LR and CR radiation are all effective for mutation induction in wheat, suggesting that CR and LR could be good mutagens for plant mutation breeding in the future. The higher mutation frequencies in this study could be partially explained by the higher LET applied in the present study, and partially by other factors such as the different species or mutant generation used in different studies.

It should be noted that RNA-seq is limited to the transcribed genetic regions, while those mutations located in the intron and non-coding regions and large size mutations are not covered. Therefore, it is understood that the calculated mutation rates in this study may be slightly lower than those based on whole genome sequencing. Whole genome sequencing may be necessary to compare the mutagenesis profiles induced by CR and LR irradiation in crops in the future.

### Uneven Chromosome Distributions of Mutations Induced by Ionizing Radiation

The distribution of IR-induced mutations on chromosomes have been discussed extensively (Li et al., 2016; Tan et al., 2019; Xiong et al., 2019; Yang et al., 2019). Fast-neutron (Li et al., 2016), gamma rays, and carbon-ion beams induced mutations in rice (Yang et al., 2019) were evenly distributed across the genome. In contrast, CR- and LR-induced mutations are biased toward one or both ends of most chromosomes, which is consistent with the previous studies in wheat that LR-induced mutations in Jing411 and Heyou1 were found on the terminal regions of chromosomes (Xiong et al., 2019). The detection method may be the potential reason of this inconsistent result. Whole genome sequencing and resequencing were used in the studies of Li et al. (2016) and Yang et al. (2019), respectively. However, RNA-seq was used in our and Xiong’s studies, which is limited to the transcribed genetic regions and associated with the distribution of genes (Figure 2). Monroe et al. (2022) revealed the natural genetic variation in mutation bias, which associates with GC content, methylated cytosines and gene structure (Monroe et al., 2022). Similarly, Weng et al. (2019) found that spontaneous mutation profile in *Arabidopsis* do not occur evenly across the genome: they are biased toward G: C to A: T transitions (Weng et al., 2019). Similar results were observed in the gamma rays, carbon-ion beams, LR, and proton beams induced mutations (Guo et al., 2019; Li et al., 2019; Xiong et al., 2019; Yang et al., 2019; Oono et al., 2020; Choi et al., 2021; Lee et al., 2021), which is also consistent with the present study. Therefore, the uneven distribution of IR induced mutations may be related to mutational bias. On the other hand, the differences between different materials suggest that genotypes may be involved in this uneven distribution, but more evidence and experiments are needed to confirm this.

### Antioxidant Processes Commonly Involved in Ionizing Radiation Response

When plants are irradiated by IR, the intracellular water is decomposed into ROS, such as superoxide anion radicals, hydroxyl radicals, hydrogen peroxide (Wang et al., 2018). A significant part (about 70–80%) of the IR-related DNA damage is caused by ROS formed during radiolysis of water and only 20–30% of the damage is due to the direct absorption of high-energy IR quanta by the target DNA molecules (Gudkov et al., 2019). To alleviate oxidative damage, plant significantly activated the antioxidant defense system using ROS detoxification of antioxidants (e.g., peroxidases and catalases) and osmotic adjustment substance (e.g., soluble sugar and proline) to maintain cellular ROS (Luo et al., 2019). For example, the exposure of barley seeds to gamma rays results in the accumulation of hydrogen peroxide in seedlings and activating the antioxidant system (Volkova et al., 2019). An RNA-seq-based study of genes differentially expressed during LR-irradiation revealed that LR-induced DEGs are associated with “phenylpropanoid biosynthesis” and “antioxidant process” (Xiong et al., 2019). Variations in phenylpropanoid and flavonoid biosynthesis pathways in response to UV-B radiation were found in date palm leaves (Maher et al., 2021).

Phenylalanine ammonia-lyase catalyzes the first step in the phenylpropanoid pathway, which plays an important role in the production of antioxidant phenolic compounds such as flavonoids and tannins. Acute gamma irradiation promoted the accumulation of the H_2_O_2_ and malondialdehyde content of rice plants, and increased the enzyme activities of phenylalanine ammonia-lyase, SOD, POD, CAT, and APX (Choi et al., 2021). Consistently, in this study, 5 phenylalanine ammonia-lyases and 16 PODs involving phenylpropanoid biosynthesis pathway were co-regulated by LR and CR.

Glucose is one of the soluble sugars in plants, which plays an important role in the plant osmotic-regulation under stresses (Khaleghi et al., 2019). The increase of soluble sugar content and the enrichment of “starch and sucrose metabolism” pathway were also reported in rice under gamma rays, cosmic rays, and carbon-ion beams treatments (Hwang et al., 2014), which is consistent with our result. The contents of soluble sugar, such as sucrose and D-glucose-6-phosphate, were increased in *Porphyra haitanensis* under UV-B exposure (Fu et al., 2021). Combined with these studies, we suggested that the accumulation of soluble sugar and the activation of ROS scavenging processes might be the important processes for plants in response to IR irradiation.

### Ionizing Radiation Type Specific Response

Under LR and CR exposure, we observed clear differences in the numbers and types of DEGs in two wheat genotypes irradiated with CR and LR (Figure 3). The number of DEG increased with the LET level, and showed a higher number of DEGs in CR treated samples than that of LR (Figure 3), implicated the type-specific responses in plants to IR irradiation exists.

In cowpea, the proton-beam treatment induced more DEGs than that of gamma-rays, and more diverse in terms of pathways were observed in the proton-beam treatment than gamma treatment. The “oxidation-reduction process” and “proteolysis” were the most enriched terms in GR treatment, and “substance metabolic process” and “cellular metabolic process” were the most enriched terms in proton-beam treatments (Kang et al., 2021). In duckweed plants, genes related to “anthocyanin accumulation” and “ATP synthesis” were specifically regulated in response to uranium treatment. While genes involved in “DNA damage and repair” and “calcium signaling” were specifically regulated in response to gamma radiation (Fu et al., 2019). In addition, the divergent response pathways in rice plants response to gamma rays, cosmic-ray, and carbon-ion beams treatments were also reported (Hwang et al., 2014), in which, genes related to “lipid metabolic process” and “gibberellin metabolic process” are greatly enriched in gamma rays treatment, while the stimulus responsive genes were significantly enriched in the carbon-ion beams irradiated plants. The molecular function pathways, such as “transcription regulator activity” and “oxidoreductase activity” were mainly enriched in the cosmic rays treatment (Hwang et al., 2014). These results suggest that plants have specific response pathways to different types of IR and that these response pathways are species-dependent (Hwang et al., 2014; Fu et al., 2019; Xiong et al., 2019; Kang et al., 2021).

Transcription factors are crucial components in signal transduction, and directly control the expression of specific sets of downstream stress-responsive genes (Luo et al., 2019). They are triggered by various signal transduction pathways and can bind to *cis*-acting elements directly or indirectly to modulate the transcription efficiency of target genes (Maher et al., 2021). In the previous study, TFs, such as MYB, WRKY, NAC, and bHLHs, were identified as regulatory proteins that are involved in regulating the expression of other genes that participate in the UV-B stress response (Maher et al., 2021). Consistently, these TFs were also detected in this study, and several IR type specific TFs were also identified between CR and LR, such as ARR-B, GRF, and HSF. Similar results were also detected in cowpeas, in which, proton-beam treatment induced more types of TFs than that of gamma-ray, and TFs, including ARR-B, B3, bZIP, C2H2, CO-like, DBB, G2-like, GRF, HSF, MYB, MYB_related, NAC, Trihelix, WOX, and WRKY, were regulated only by the proton beam when compared with gamma rays treatment (Kang et al., 2021). In rice, ARR-B and PHOR1 TFs were specifically induced by carbon-ion beams and gamma rays, respectively (Hwang et al., 2014). The results indicated that plants complexly regulated by varying the combination and concentration of TFs according to the IR and the crop species.

### Distinct DNA Methylation Alterations Induced by CR and LR

Plant response to DNA damage through epigenetic modifications has been well documented (Kim, 2019). Epigenetic mechanisms, such as histone modification and DNA methylation, involving response to radiation, have also been demonstrated (Shi et al., 2014; Georgieva et al., 2017; Pan et al., 2017; Zhao et al., 2018; Horemans et al., 2019; Marfil et al., 2019). Gamma rays have been shown to cause local or global changes in the chromatin structure, including an active (H4K12ac, H3K36me3, H3K4me3, H3K4ac, and H3K27ac) or repressive (H3K27me3) chromatin state (Pan et al., 2017). In addition, chernobyl radio-contaminated Pine trees (Volkova et al., 2018) and soybean seedlings (Georgieva et al., 2017) showed an increase in global DNA methylation, while a significant decrease in *Arabidopsis* (Horemans et al., 2018). UV-B induced hypermethylation in the grapevine (Marfil et al., 2019), while hypomethylation in sweet wormwood (Pandey and Pandey-Rai, 2015). In this study, DNA methylation levels increased in both wheat genotypes under LR treatment, while decreased under CR treatment. These contrasting reports imply that different methylation mechanisms for radiation response may exist in plant species.

Further analysis of DNA methylation patterns showed that DNA hypomethylation and hypermethylation are concurrent in the both LR and CR treatments, with LR inducing a higher rate of DNA hypermethylation than hypomethylation. Notably, this pattern was reversed under CR irradiation. Similar results were reported in rice under carbon-ion radiation (Zhao et al., 2018), which showed that low-dose carbon-ion radiation (0.01, 0.2, or 1 Gy) induced higher proportion of hypermethylation than hypomethylation, whereas high-dose carbon-ion radiation (2, 5, or 20 Gy) induced more hypomethylation than hypermethylation. These results suggest that the epigenetic regulation patterns were quite complex and may vary across species depending on the different types of IRs.

## Conclusion

The present study showed that both LR and CR are high efficiency mutagens in wheat. Both LR and CR induced higher number of SBSs than InDels. The mutations were unevenly distributed on the wheat chromosome. Comparative analysis showed that CR induced more mutations than LR. Transcriptomic analysis suggested that antioxidant processes were commonly pathways of wheat in response to LR and CR irradiation. IR type specific TFs and response pathways were detected. The DNA methylation levels were increased under LR irradiation, while decreased under CR irradiation. LR induced higher proportion of hypermethylation than hypomethylation, whereas CR induced higher proportion of hypomethylation than hypermethylation. The genomic and epigenetic characterization induced by CR and LR enhanced our knowledge of the mechanism of mutagenesis and mutation breeding in wheat. In the future, track the transmission of the variations between generations is the key problem for breeding utilization.

## Data Availability Statement

The datasets presented in this study can be found in online repositories. The name of the repository and accession number can be found below: SRA, NCBI; PRJNA805296 (https://www.ncbi.nlm.nih.gov/bioproject/PRJNA805296).

## Author Contributions

YX, CJ, and LL designed the experiments. LZ and LL treated the samples. BL, LX, BA, and RW conducted the research. BL, LZ, SZ, HC, YH, and GL analyzed the results. BL, LZ, and YX wrote the whole manuscript. YX, BL, CJ, and LL revised the manuscript. All authors contributed to the article and approved the submitted version.

## Conflict of Interest

The authors declare that the research was conducted in the absence of any commercial or financial relationships that could be construed as a potential conflict of interest.

## Publisher’s Note

All claims expressed in this article are solely those of the authors and do not necessarily represent those of their affiliated organizations, or those of the publisher, the editors and the reviewers. Any product that may be evaluated in this article, or claim that may be made by its manufacturer, is not guaranteed or endorsed by the publisher.

## Footnotes

1
http://www.wheatgenome.org

2
https://software.broadinstitute.org/gatk

3
http://www.omicshare.com/

4
http://planttfdb.gao-lab.org/

5
http://jvenn.toulouse.inra.fr/app/index.html

## Supplementary Material

Supplementary Figure 1 Correlation analysis between biological replicates. The horizontal axis and vertical axis represent each sample. The color represents the correlation coefficient, the redder the color, the higher the correlation, and the whiter the color, the lower the correlation.

Supplementary Figure 2 The expression pattern of 15 selected genes identified by RNA-seq was verified by qRT-PCR. Heat map showing the expression changes (log2-fold change) in response to the LR and CR treatments for each candidate gene as measured by RNA-seq **(A)** and RT-PCR **(B)**. **(C)** Correlation analysis of differentially expressed genes between RT-PCR analysis and RNA-seq experiment. All treatments were set three biological and three technical replications.

Supplementary Figure 3 Partial MSAP bands monitored by capillary gel electrophoresis. H and M represent genomic DNA sample digested by *Eco*RI/*Hpa*II and *Eco*RI/*Msp*I combinations, respectively. MSAP markers generated with the selected primer combinations of *Hpa*II/*Msp*I + CTG/*Eco*RI + GTT (primer combination 1), *Hpa*II/*Msp*I + CTG/*Eco*RI + AAC (primer combination 2), and *Hpa*II/*Msp*I + TCCA/*Eco*RI + TA (primer combination 3).
