# Supplementary figures and images for: The Mutational, Epigenetic, and Transcriptional Effects Between Mixed High-Energy Particle Field (CR) and 7Li-Ion Beams (LR) Radiation in Wheat M1 Seedlings
Source: Front Plant Sci. 2022 May 11;13:878420. doi: 10.3389/fpls.2022.878420 (PMC9131052; doi:10.3389/fpls.2022.878420)

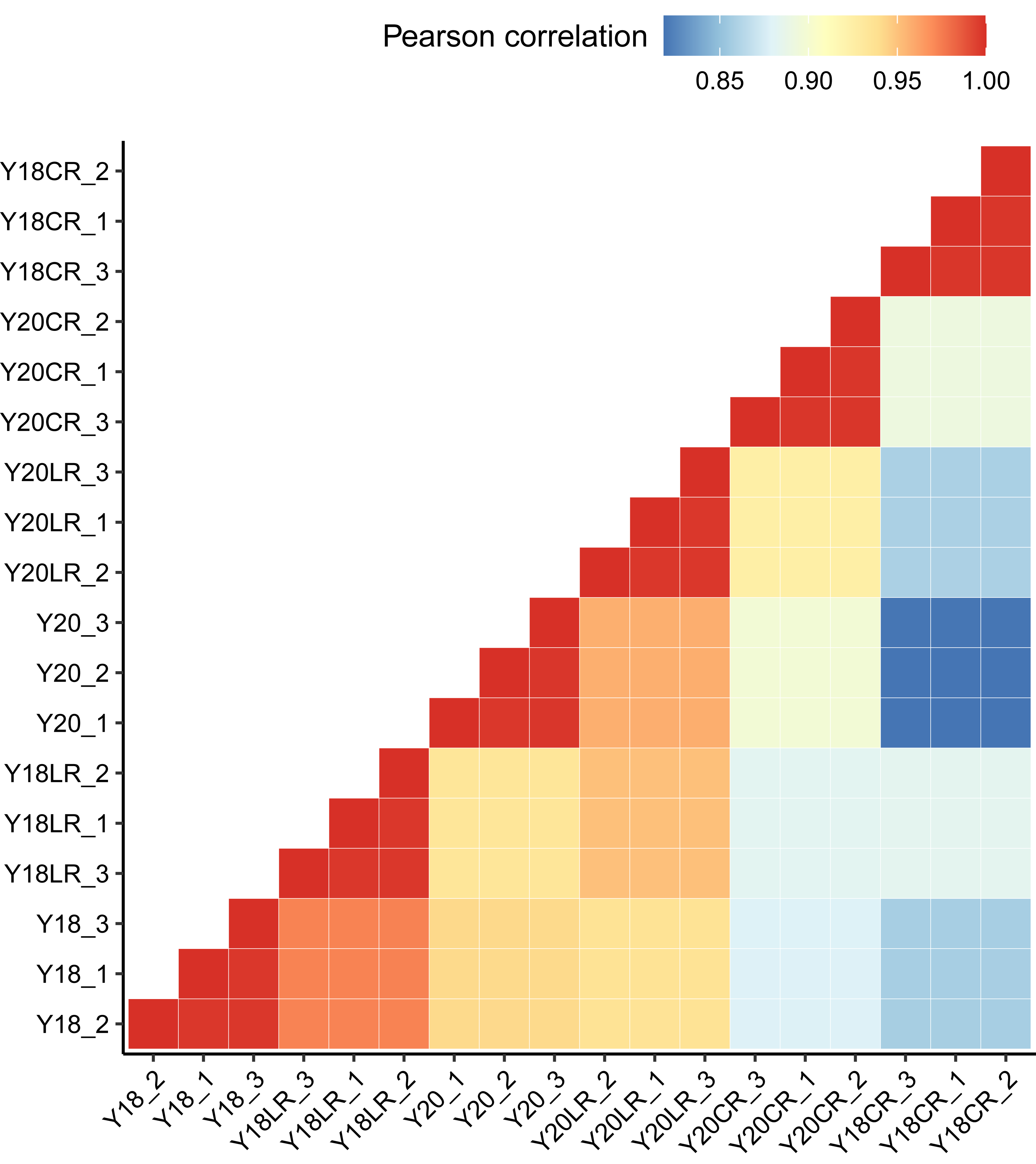

Supplement: Supplementary Figure 1 — Correlation analysis between biological replicates. The horizontal axis and vertical axis represent each sample. The color represents the correlation coefficient, the redder the color, the higher the correlation, and the whiter the color, the lower the correlation. [file Image_1.TIF]

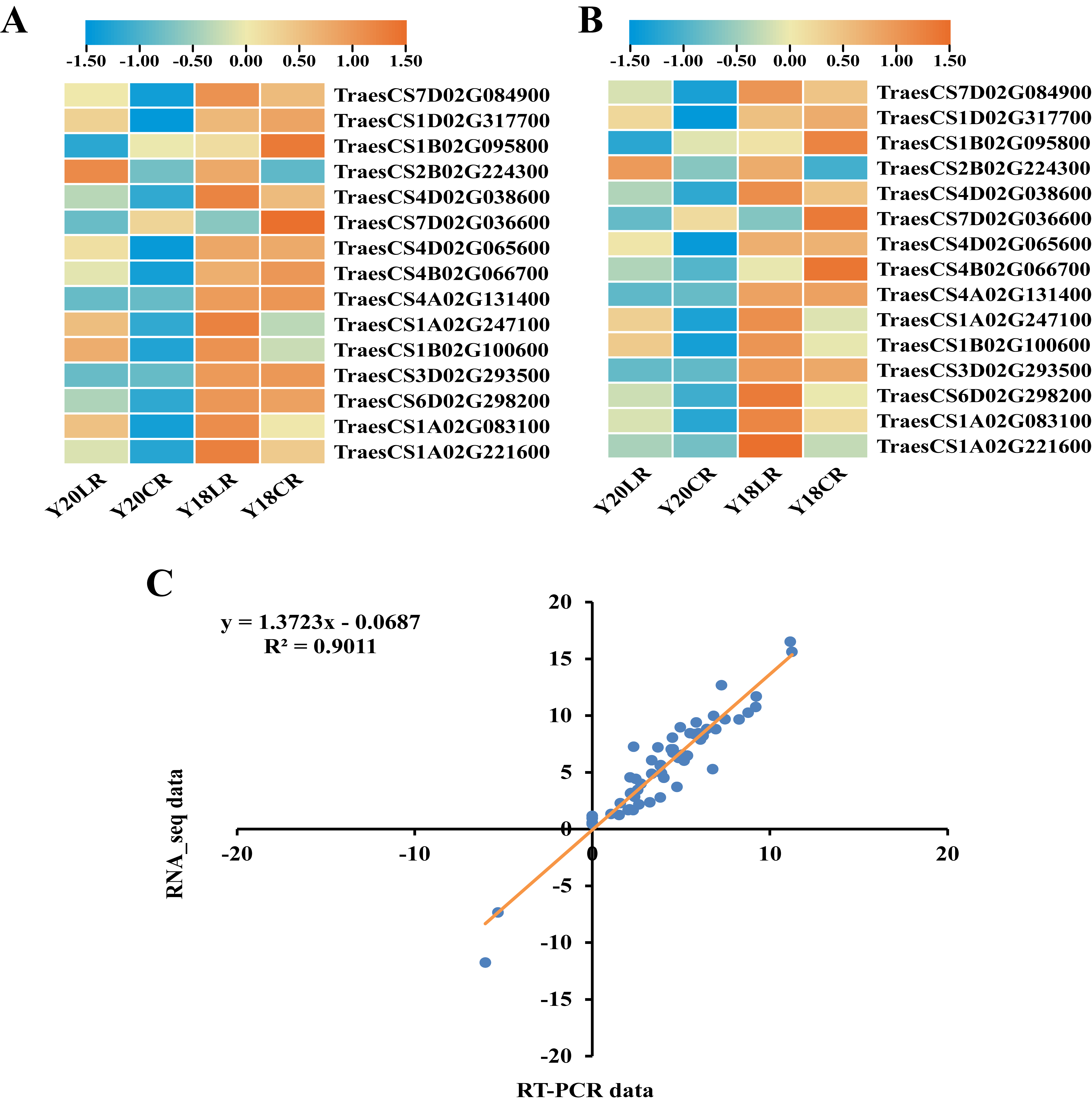

Supplement: Supplementary Figure 2 — The expression pattern of 15 selected genes identified by RNA-seq was verified by qRT-PCR. Heat map showing the expression changes (log2-fold change) in response to the LR and CR treatments for each candidate gene as measured by RNA-seq (A) and RT-PCR (B). (C) Correlation analysis of differentially expressed genes between RT-PCR analysis and RNA-seq experiment. All treatments were set three biological and three technical replications. [file Image_2.TIF]

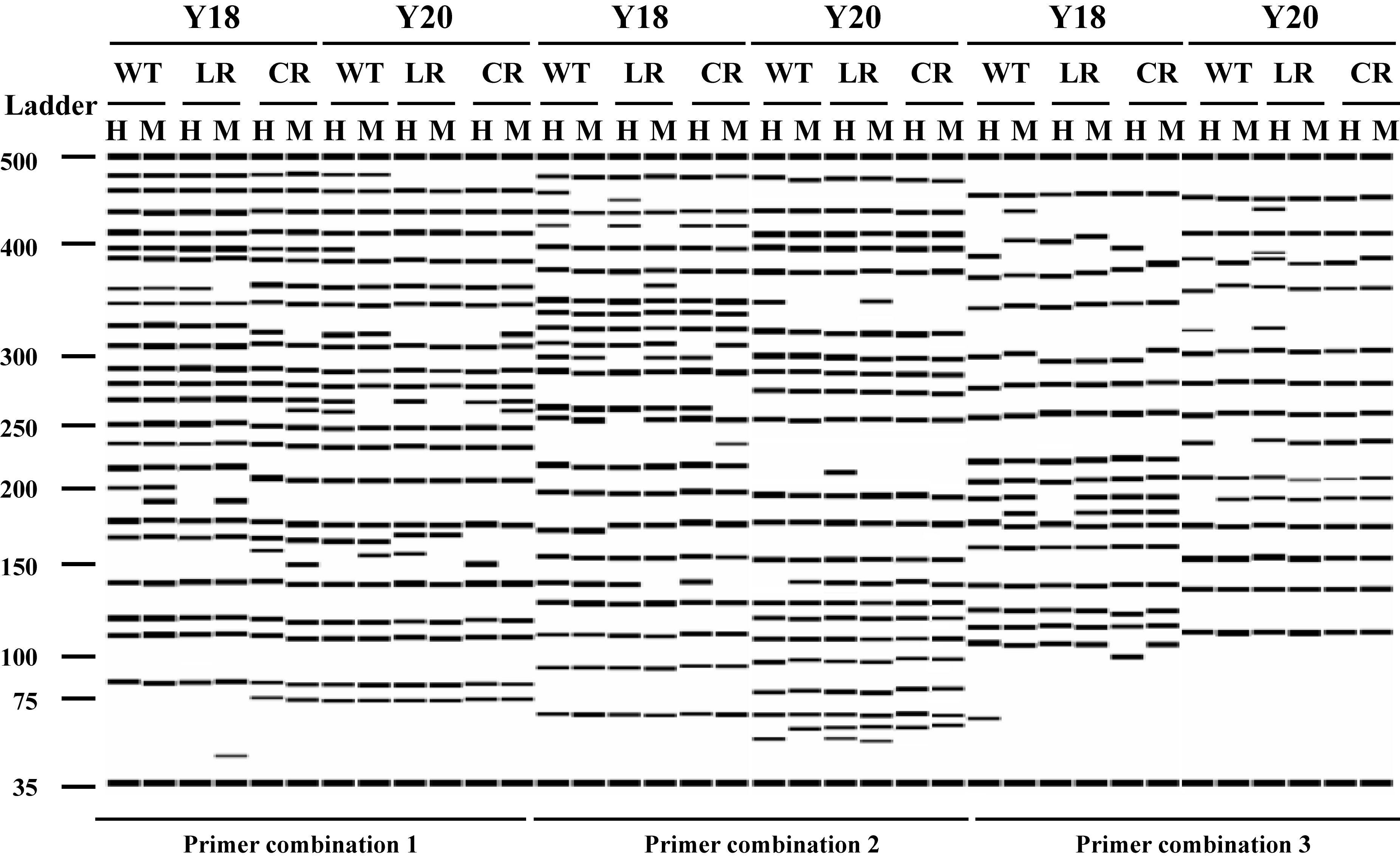

Supplement: Supplementary Figure 3 — Partial MSAP bands monitored by capillary gel electrophoresis. H and M represent genomic DNA sample digested by EcoRI/HpaII and EcoRI/MspI combinations, respectively. MSAP markers generated with the selected primer combinations of HpaII/MspI + CTG/EcoRI + GTT (primer combination 1), HpaII/MspI + CTG/EcoRI + AAC (primer combination 2), and HpaII/MspI + TCCA/EcoRI + TA (primer combination 3). [file Image_3.TIF]
